# Molecular genetic diagnostics of hypogonadotropic hypogonadism: from panel design towards result interpretation in clinical practice

**DOI:** 10.1007/s00439-020-02148-0

**Published:** 2020-03-28

**Authors:** Henriett Butz, Gábor Nyírő, Petra Anna Kurucz, István Likó, Attila Patócs

**Affiliations:** 1 Department of Laboratory Medicine, Semmelweis University, Nagyvárad tér 4, Budapest, 1089 Hungary; 2 Hereditary Tumours Research Group, Hungarian Academy of Sciences and Semmelweis University, Budapest, Hungary; 3 Department of Molecular Genetics, National Institute of Oncology, Budapest, Hungary; 4 Molecular Medicine Research Group, Hungarian Academy of Sciences and Semmelweis University, Budapest, Hungary; 5 2nd Department of Internal Medicine, Semmelweis University, Budapest, Hungary

## Abstract

Congenital hypogonadotropic hypogonadism (CHH) is a clinically and genetically heterogeneous congenital disease. Symptoms cover a wide spectrum from mild forms to complex phenotypes due to gonadotropin-releasing hormone (GnRH) deficiency. To date, more than 40 genes have been identified as pathogenic cause of CHH. These genes could be grouped into two major categories: genes controlling development and GnRH neuron migration and genes being responsible for neuroendocrine regulation and GnRH neuron function. High-throughput, next-generation sequencing (NGS) allows to analyze numerous gene sequences at the same time. Nowadays, whole exome or whole genome datasets could be investigated in clinical genetic diagnostics due to their favorable cost–benefit. The increasing genetic data generated by NGS reveal novel candidate genes and gene variants with unknown significance (VUSs). To provide clinically valuable genetic results, complex clinical and bioinformatics work are needed. The multifaceted genetics of CHH, the variable mode of inheritance, the incomplete penetrance, variable expressivity and oligogenic characteristics further complicate the interpretation of the genetic variants detected. The objective of this work, apart from reviewing the currently known genes associated with CHH, was to summarize the advantages and disadvantages of the NGS-based platforms and through the authors’ own practice to guide through the whole workflow starting from gene panel design, performance analysis and result interpretation. Based on our results, a genetic diagnosis was clearly identified in 21% of cases tested (8/38).

**Electronic supplementary material:**

The online version of this article (10.1007/s00439-020-02148-0) contains supplementary material, which is available to authorized users.

## Congenital hypogonadotropic hypogonadism (CHH)

### Congenital hypogonadotropic hypogonadism (CHH) as a clinically heterogeneous entity

Congenital hypogonadotropic hypogonadism (CHH) is a genetic condition characterized by incomplete or absent puberty and infertility due to central (tertiary or hypothalamic) hypogonadism caused by gonadotropic hormone-releasing hormone (GnRH) deficiency. Three clinical forms are distinguished by the European consensus: (1) GnRH deficiency with defective sense of smell (Kallmann syndrome, KS), (2) isolated GnRH deficiency (normosmic CHH) and the third form when KS/CHH is part of a complex genetic syndrome (Boehm et al. 2015). CHH has an incidence of 1:125,000 in female and 1:30,000 in males indicating the male predominance (Stamou and Georgopoulos 2018). CHH has a heterogeneous clinical appearance, and lately the constitutional delay of growth and puberty (CDGP), the adult-onset hypogonadotropic hypogonadism and the hypothalamic amenorrhea are also considered as a milder end of the spectrum (Stamou and Georgopoulos 2018). Most patients are diagnosed in adolescence due to delayed puberty. In male, neonate cryptorchidism and micropenis can be considered as signs of CHH, but there are no specific signs of CHH in female neonates (Young et al. 2019). Prepubertal testes and undervirilized secondary sexual features are the most common symptoms in males, while absence of breast development and primary amenorrhea occur in females as a consequence of CHH (Young et al. 2019). The disease can be diagnosed in adulthood as well by low libido, infertility, bone loss and fractures when it is untreated (Young et al. 2019). Interestingly, in 10–20% of the cases, CHH is reported reversible, however, the pathophysiology behind this is not clearly revealed (Stamou and Georgopoulos 2018; Young et al. 2019). To establish the biochemical diagnosis in infants is challenging as GnRH neurons are active only during mini-puberty (4–8 weeks after birth) and after that, their activity becomes quiescent until puberty. In adolescence, results of biochemical tests (basal and stimulated blood levels of sex hormones and gonadotropins), brain imaging for examination of olfactory bulbs, assessment of smell and evaluation of family history are parts of the routine medical investigations. (Naturally, additional work-ups, i.e. evaluation of bones, kidneys and sexual organs, are also required for diagnosis and for differential diagnostic purposes (Young et al. 2019). Constitutional delayed of growth and puberty (CDGP) is defined as the lack of the start of sexual maturation at an age > 2 SDs above the mean for a given population (Stamou and Georgopoulos 2018). There is no identifiable cause behind and finally puberty occurs. 50–80% of CDGP individuals have positive family history of the phenomenon, and approximately 10% of CHH patients have relatives with CDGP (Stamou and Georgopoulos 2018). Differentiating CDGP and CHH in adolescence is challenging as to date no gold-standard diagnostic test is known for this purpose (Young et al. 2019).

There are non-reproductive features as well that are commonly recognized in patients with CHH. Midline facial defects (cleft lip or palate), dental agenesis, unilateral renal agenesis, short metacarpals, hearing loss, synkinesia, cerebellar ataxia can appear additionally to CHH (Young et al. 2019). Furthermore, the disease can occur as part of complex genetic syndromes summarized in Table 1.

**Table 1:** Congenital hypogonadotropic hypogonadism gene characteristics

Gene name	Inheritance	Gene function contributing in symptoms	Clinical phenotype based on expert consensus (Boehm et al. 2015) and OMIM database	OMIM number ID or Ref
ANOS1 (KAL1)	XR, contributes to oligogenicity	Migration of GnRH neurons	Kallmann syndrome, CHH reversal	308700
AXL	AD	Migration of GnRH neurons	Kallmann syndrome; CHH	Salian-Mehta et al. (2014)
BBS1, BBS2, ARL6, BBS4, BBS5, MKKS, BBS7, TTC8, BBS9, BBS10, TRIM32, BBS12	AD or AR	Maintenance and function of cilia (cell movement, perception of sensory input such as sight, hearing, and smell)	Bardet-Biedl syndrome 1–12	209900
CHD7	AD	Migration of GnRH neurons	Kallmann syndrome; CHH with or without reversal; CHARGE sy	612370
CPE	AR	As part of a syndrome	CHH, obesity, diabetes mellitus type 2	Alsters et al. (2015)
DCAF17	AR	As part of a syndrome	Woodhouse-Sakati syndrome	241080
DMXL2	AD or AR	As part of a syndrome	CHH; PEPNS (polyendocrine deficiencies and polyneuropathies)	616113
DUSP6	AD	Migration of GnRH neurons	CHH with or without anosmia	615269
FEZF1	AR	Migration of GnRH neurons	Kallmann syndrome	616030
FGF17	AD, contributes to oligogenicity	Hypothalamus/pituitary development	Kallmann syndrome; CHH; Dandy-Walker sy	615270
FGF8	AD, contributes to oligogenicity	Hypothalamus/pituitary development,migration of GnRH neurons, neuroendocrine regulation	Kallmann syndrome; CHH; Combined pituitary hormone deficiency	612702
FGFR1	AD, contributes to oligogenicity	Hypothalamus/pituitary development, neuroendocrine regulation	Kallmann syndrome; CHH with or without reversal; Combined pituitary hormone deficiency; Septo-optic dysplasia; Hartsfield sy; Split hand/foot malformation	147950
FLRT3	AD	Migration of GnRH neurons	CHH with anosmia	615271
FSHB	AR	Hypothalamus/pituitary development	CHH without anosmia	229070
GLCE	AD, AR	Hypothalamus/pituitary development	KS, CHH	Stamou and Georgopoulos (2018)
GNRH1	AR	neuroendocrine regulation	CHH	614841
GNRHR	AR, contributes to oligogenicity	Neuroendocrine regulation	CHH with or without reversal	146110
HDAC8	XR	Transcriptional regulation, cell cycle progression and development	Cornelia de Lange syndrome 5	300882
HESX1	AD or AR	Hypothalamus/pituitary development	Kallmann syndrome; Combined pituitary hormone deficiency, Septo-optic dysplasia	182230
HFE	AD or AR	Controlling iron absorption by regulating the interaction of the transferrin receptor with transferrin	Hereditary hemochromatosis	235200
HS6ST1	AD, contributes to oligogenicity	Migration of GnRH neurons	Kallmann syndrome; CHH with or without reversal	614880
IL17RD (SEF)	AD, AR or digenic dominant, contributes to oligogenicity	Migration of GnRH neurons	CHH with or without anosmia	615267
IRF2BPL (EAP1)	Not known	Neuroendocrine regulation	CHH	Mancini et al. (2019)
KISS1	AR	neuroendocrine regulation	CHH	614842
KISS1R	AR, contributes to oligogenicity	Neuroendocrine regulation	CHH	614837
LEP	AR	Neuroendocrine regulation	CHH, obesity	Strobel et al. (1998)
LEPR	AR	Neuroendocrine regulation	CHH, obesity	Hannema et al. (2016)
LHB	AR	Hypothalamus/pituitary development	CHH with or without anosmia	228300
LHX3	AR	Hypothalamus/pituitary development	Combined pituitary hormone deficiency	221750
NDN	AD	Neuroendocrine regulation	Prader-Willi syndrome	176270
NR0B1 (DAX1)	XR	Hypothalamus/pituitary development	CHH, congenital adrenal hypoplasia	300200
NSMF (NELF)	AD, contributes to oligogenicity	Migration of GnRH neurons	Kallmann syndrome; CHH with or without reversal	614838
OTUD4	AR	As a deubiquitinase it negatively regulates inflammatory and pathogen recognition signaling in innate immune response	CHH; Gordon-Holmes sy	212840
PCSK1	AR	Hypothalamus/pituitary development	CHH, obesity	600955
PNPLA6	AR	Regulation of neurite outgrowth and process elongation during neuronal differentiation	CHH; Boucher-Neuhauser syndrome	215470
POLR3A	AR	As a DNA-dependent RNA polymerase it catalyzes the transcription of DNA into RNA	Leukodystrophy, hypomyelinating, seven, with or without oligodontia and/or hypogonadotropic hypogonadism	607694
POLR3B	AR	As a DNA-dependent RNA polymerase it catalyzes the transcription of DNA into RNA	Leukodystrophy, hypomyelinating, eight, with or without oligodontia and/or hypogonadotropic hypogonadism	614381
PROK2	AD, contributes to oligogenicity	Migration of GnRH neurons	Kallmann syndrome; CHH	610628
PROKR2	AD, contributes to oligogenicity	Migration of GnRH neurons	Kallmann syndrome; CHH with or without reversal; Combined pituitary hormone deficiency; Morning glory syndrome	244200
PROP1	AR	Hypothalamus/pituitary development	Combined pituitary hormone deficiency	262600
RAB18	AR	Eye and brain development, regulating membrane trafficking in organelles and transport vesicles	Warburg micro syndrome 3	614222
RAB3GAP1	AR	Eye and brain development, regulated exocytosis of neurotransmitters and hormones	Warburg micro syndrome 1	600118
RAB3GAP2	AR	Eye and brain development, regulated exocytosis of neurotransmitters and hormones	Martsolf syndrome (cataract, mental-retardation, hypogonadism)	212720
RBM28	AR	Splicing regulator	Alopecia, neurologic defects, and endocrinopathy syndrome	612079
RNF216	AR	Ubiquitination, protein degradation by the proteasome, regulation of TNF-, IL1- and NFKB signaling	CHH; Gordon-Holmes sy	212840
SEMA3A	AD, contributes to oligogenicity	Migration of GnRH neurons	Kallmann syndrome	614897
SEMA3E	AD	Migration of GnRH neurons	KS, CHH	Cariboni et al. (2015)
SEMA7A	contributes to oligogenicity	Migration of GnRH neurons	Kallmann syndrome; CHH	Känsäkoski et al. (2014)
SOX10	AD	Hypothalamus/pituitary development	Kallmann syndrome; Waardenburg syndrome; PCWH syndrome (peripheral demyelinating neuropathy, central demyelination, Waardenburg syndrome, and Hirschsprung disease)	611584
SOX2	AD	Hypothalamus/pituitary development	CHH, optic nerve hypoplasia and abnormalities of the central nervous system	206900
SOX3	XR	Hypothalamus/pituitary development	CHH	Izumi et al. (2014)
SPRY4	AD	Migration of GnRH neurons	Kallmann syndrome	615266
SRA1	AD	Neuroendocrine regulation	CHH	Kotan et al. (2016)
STUB1	AR	Ubiquitination, protein degradation by the proteasome	Spinocerebellar Ataxia 16, Autosomal Recessive	615768
TAC3	AR, contributes to oligogenicity	Neuroendocrine regulation	CHH with or without reversal	614839
TACR3	AR, contributes to oligogenicity	Neuroendocrine regulation	CHH with or without reversal	614840
TBC1D20	AR	Regulation of vesicle-mediated transport, GTPase-activating protein specific for Rab1 and Rab2	Warburg micro syndrome 4	615663
TUBB3	AD	Migration of GnRH neurons	“TUBB3 E410K syndrome”	Chew et al. (2013); Patel et al. (2017)
WDR11	AD, contributes to oligogenicity	Migration of GnRH neurons	Kallmann syndrome; CHH with or without reversal; Combined pituitary hormone deficiency	614858

Diagnostics and genetic counseling is important in CHH as effective therapies are available for the development of secondary sexual features and fertility (Maione et al. 2018; Young et al. 2019).

Adult onset of hypogonadotropic hypogonadism is a rare form of CHH. It is a non-reversible, long-lasting condition but the etiology and pathogenesis have to be investigated and demonstrated. The diagnosis can be made when all other acquired causes of hypogonadotropic hypogonadism (e.g. structural anomalies, infiltrative/inflammatory origin, pituitary/CNS tumors etc.) have been excluded (Stamou and Georgopoulos 2018).

### Genetic background of CHH

CHH is heterogeneous not only clinically but also genetically. To date, more than 40 genes have been identified as pathogenic cause in the background of the disease (Boehm et al. 2015; Maione et al. 2018; Stamou and Georgopoulos 2018). Analysis the individual CHH genes (Table 1) one by one exceeds the goal of our study, but these are excellently reviewed in recent papers (Topaloglu and Kotan 2016; Topaloğlu 2018; Maione et al. 2018). Genes implicated in the pathogenesis of CHH are usually divided into two major categories (Boehm et al. 2015; Topaloğlu 2018; Maione et al. 2018; Stamou and Georgopoulos 2018). The first group consists of genes that control development and GnRH neuron migration. Therefore, the pathogenic variants of these genes are frequently associated with anosmia and midline developmental anomalies (Table 1). The second group of genes is responsible for neuroendocrine physiology and GnRH neuron function (either by afferent modulators or by regulating GnRH secretion), these can be detected in normosmic CHH forms. Although there are genes with multiple roles that participate in both mechanisms, their mutations can be often identified in both anosmic and normosmic forms (Boehm et al. 2015; Maione et al. 2018; Stamou and Georgopoulos 2018) (Table 1).

Autosomal dominant, autosomal recessive and X-linked inheritance have been identified, however, with the availability of high-throughput next-generation sequencing at least 20% of CHH cases have thought to be di- or oligogenic. In these cases, two or more gene variants can be identified in the same patient (Boehm et al. 2015) (Table 1). Still, in more than half of the CHH cases, there is no pathogenic mutation identified. Among the main genetic forms of CHH, the most common autosomal recessively inherited types are caused by *GNRHR*, *KISS1R* and *TACR3* variants (Maione et al. 2018). Kallmann syndrome caused by *ANOS1* gene mutations is inherited by X-linked recessive trait as it is located on chromosome X. *FGFR1* and *PROK2*/*PROKR2* lead to autosomal dominantly inherited type of CHH (Boehm et al. 2015; Maione et al. 2018). Regarding *FGFR1,* nearly half, regarding *PROK2*/*PROKR2,* nearly two-third of the cases exhibit incomplete penetrance and variable expressivity that complicate the determination of inheritance (Maione et al. 2018). Recently, a normosmic CHH patient was reported who inherited a pathogenic variant in *GNRHR* gene in a homozygous form due to the occurrence of uniparental isodisomy (Cioppi et al. 2019). (Uniparental disomy-UPD is a non-Mendelian inheritance pattern when an individual has inherited two copies of a specific chromosome (or part of it) from a single parent. When a chromosomal pair inherited from the same parent, it is called uniparental heterodisomy, when two identical chromosomes are inherited it is called uniparental isodisomy (iUPD). This discovery further complicates the inheritance pattern of CHH and raises the possibility of the same phenomenon in case of other genes as well.

Individual relevance of genes in oligogenic CHH cases are needed to be interpreted with cautions. For instance, another candidate CHH gene *NSMF* (earlier *NELF*), listed in the expert consensus statement, has now a controversial role (Spilker et al. 2016). Several publications reported *NSMF* variants in CHH patients alone or in combination with a mutation in another gene (Miura et al. 2004; Pitteloud et al. 2007; Xu et al. 2011) underlining again its questionable role (Spilker et al. 2016).

Interestingly, rare variants of *TAC3*, *TACR3* and other genes are suggested to be linked with CHH reversal that further raises the possibility of therapy discontinuation from time to time to test the reversibility of CHH in these carriers (Gianetti et al. 2010; Boehm et al. 2015).

Variants of known CHH genes have been investigated and identified in CDGP and in cases with hypothalamic amenorrhea too. This suggests that the time of menarche and menopause are genetically determined which is strongly supported by family histories (Stamou and Georgopoulos 2018).

In CDGP, Zhu et al. identified that variants in CHH genes were enriched in CDGP family members compared to unaffected family members suggesting the genetic link between CHH and CDGP (Zhu et al. 2015). This is further supported by variants identified in CDGP patients in *TAC3*, *TACR3*, *IL17RD*, *GNRHR*, *PROKR2*, *HS6ST1*, *FGFR1*, *FEZF1*, *AXL* genes (Gianetti et al. 2012; Tusset et al. 2012; Zhu et al. 2015; Hietamäki et al. 2017; Cassatella et al. 2018). However, results of Cassatella et al. demonstrated that CDGP and CHH have distinct genetic profiles that may facilitate the differential diagnosis in patients presenting with delayed puberty (Cassatella et al. 2018).

Hypothalamic amenorrhea is also a reversible dysfunctional feature that can be triggered by nutritional deficit, extensive exercise or psychological stress. Genetic variants have been identified in *FGFR1*, *PROKR2*, *GNRHR* and *ANOS1* genes suggesting that these mutations may contribute to the variable functional changes in GnRH secretion (Caronia et al. 2011).

CHH can be also part of complex genetic syndromes which are summarized by Boehm et al. and genetic background are summarized in Table 1 (Boehm et al. 2015).

### Genetic testing and genetic counseling in CHH

#### Testing strategies

**Fig. 1:**
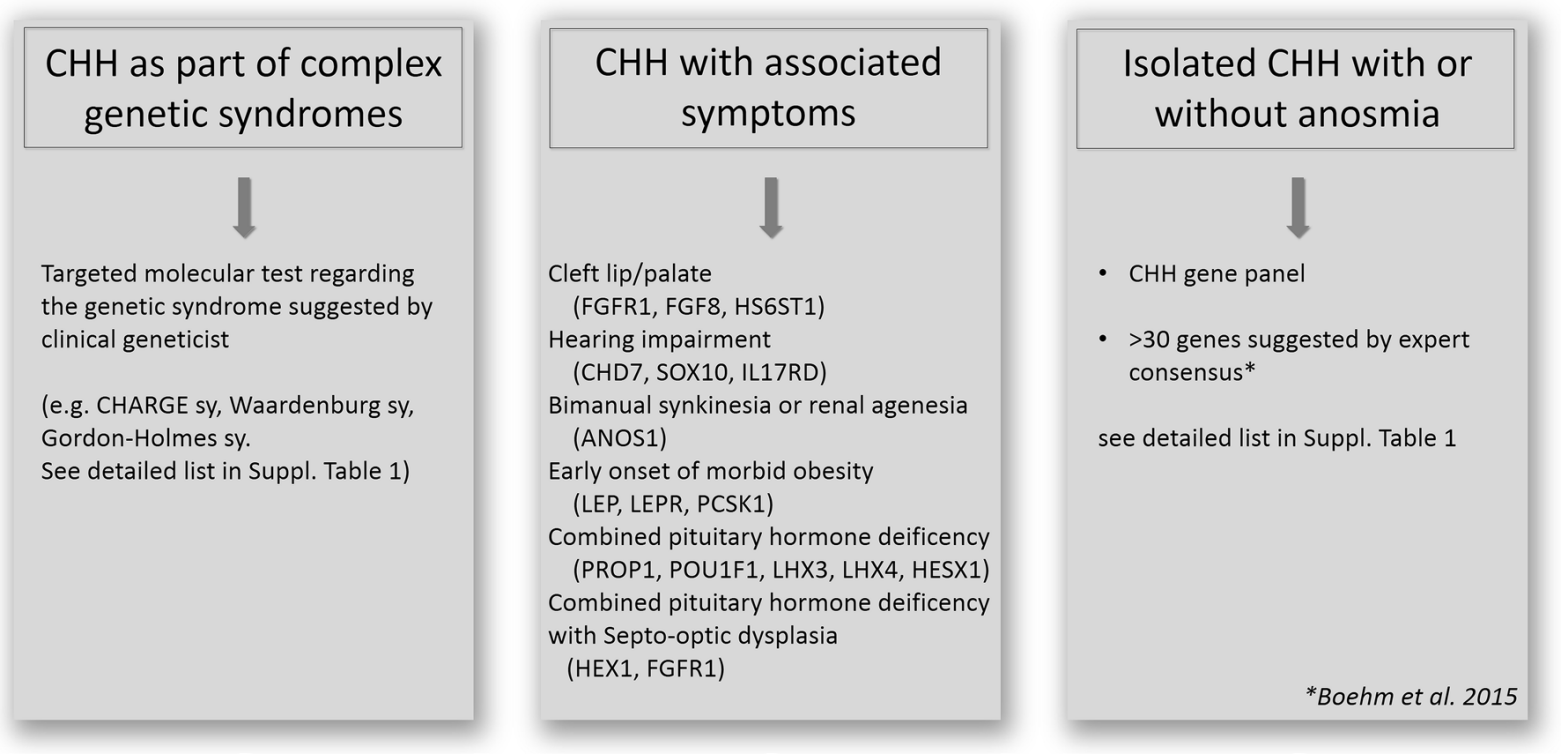
Genetic testing strategies in CHH (based on Boehm et al. 2015; Stamou and Georgopoulos 2018; Topaloğlu 2018)

Although high-throughput screening can be recommended, targeted panel testing, prioritization and gene selection based on clinical data are also possible (Boehm et al. 2015; Topaloğlu 2018; Stamou and Georgopoulos 2018). The first step is to exclude the presence of genetic syndromes based on clinical findings. When a clinical geneticist based on the whole clinical presentation indicates a specific syndrome (e.g. CHARGE sy., Bardet-Biedl sy., Gordon-Holmes sy., see details in Table 1) targeted gene testing is recommended. When complex syndromes can be excluded additional associated signs and symptoms can increase the probability of finding casual mutations (Boehm et al. 2015). For instance, besides anosmia/hyposmia, bimanual synkinesia or renal agenesis can associate with *ANOS1* mutation (Fig. 1). Cleft palate/lip, dental agenesis and digital bone anomalies were frequently associated with CHH caused by mutations in genes of FGF8 signaling (*FGFR1*, *FGF8*, *HS6ST1*) (Costa-Barbosa et al. 2013; Boehm et al. 2015). Hearing impairment commonly appeared with CHH in *CHD7*, *SOX10* or *IL17RD* mutation carriers (Costa-Barbosa et al. 2013; Boehm et al. 2015). Additionally, early onset of morbid obesity with CHH could suggest variants in *LEP*, *LEPR* or *PCSK1* genes (Jackson et al. 1997; Farooqi and O’Rahilly 2008). If CHH is associated with severe adrenal insufficiency congenital adrenal hypoplasia caused by *NR0B1* (*DAX1*) is likely.

Combined pituitary hormone deficiency (CHPD) should also be clinically investigated/excluded as CHH and CHPD have overlapping genetic etiologies. If isolated CHPD is diagnosed, genetic testing of genes encoding the pituitary transcription factors (*PROP1*, *POU1F1*, *LHX4*, *LHX3* and *HESX1*) should be recommended (Fang et al. 2016). However, lately, variants of certain CHH genes including *CHD7*, *PROKR2*, *WDR11*, *FGFR1* and *FGF8* have also been implicated in CPHD (Raivio et al. 2012; Fang et al. 2016). Similar to CHH, CPHD is suggested to be a multifactorial disease as symptoms frequently present incomplete penetrance even harboring the very same mutations (Raivio et al. 2012).

Genetic testing starts with evaluation of the inheritance pattern using pedigree analysis. However, Mendelian inheritance have been described for the majority of genes associated with CHH, some genes show different inheritance patterns (e.g. *FGFR1*: AD/AR/oligogenic/de novo; *PROK2*/*PROKR2*: AD/AR/oligogenic), see Table 1 (Boehm et al. 2015; Maione et al. 2018).

Parallel with revolution of molecular genetic technologies for patients with CHH multi-gene panel testing can be recommended, because there is a wide overlap between both symptoms and genetic background (Boehm et al. 2015; Maione et al. 2018). Since expert consensus have been published in 2015 (Boehm et al. 2015) several high-throughput multi-gene panel studies were carried out. However, there is no consented gene list that should be offered for patients providing an accurate diagnosis for the majority of cases. After publication of the expert consensus 7 CHH gene panel testing studies were reported (Table 2) (Quaynor et al. 2016; Aoyama et al. 2017; Wang et al. 2017; Cassatella et al. 2018; Zhou et al. 2018; Amato et al. 2019; Kim et al. 2019). In these studies, 25–261 genes were included as susceptibility genes of CHH. The positive detection rate varied between 33 and 56% (Table 2). Mutations in the *FGFR1* gene were found the most commonly (in all eight studies), *ANOS1* (in seven studies) and *CHD7*, *PROKR2*, *TACR3* and *IL17RD* variants were also frequently detected (in six studies) among different groups (Table 2). Analyzing the detection rate by patient number *FGFR1* variants were detected most commonly, in an average of 11.4% of all investigated patients, *CHD7, PROKR2* and *ANOS1* in 8.4, 6.4 and 5.7% of patients, respectively, across all studies. All other gene variants were found less than an average of 3% in these patients (see details in Table 2). Di- and oligogenic cases occurred approximately between 10 and 20% of all cases.

**Table 2:** High-throughput NGS studies investigating CHH patients

	Quaynor et al. (2016)	Wang et al. (2017)	Aoyama et al. (2017)	Cassatella et al. (2018)		Zhou et al. (2018)	Kim et al., (2019)	Amato et al. (2019	Current study
Nr of CHH patients	48	51	22	116 CHH	72 CDGP	153	28	130	38
Nr of genes investigated	261	164	27	25		83	69	36	41
Nr of patients with identified variant	NA	26 (51%)	12 (54.5%)	59 (51%)	5 (7%)	87 (56%)	11 (39%)	43 (33%)	22 (57%)
Ratio of cases with di-/oligogenic background	19%	9.8%	0%	15%	1.4%	19%	not reported	6.9%	21%
Genes with variants identified (frequency of detection in the partciular cohort)	AXL (6.3%)	PROKR2 (17.6%)	CHD7 (18.2%)	FGFR1 (15.5%)	AXL (1.4%)	RELN (20.3%)	FGFR1 (14.3%)	CHD7 (10.8%)	FGFR1 (12.5%)
	FGFR1 (6.3%)	FGFR1 (13.7%)	ANOS1 (18.2%)	CHD7 (13.8%)	FGFR1 (1.4%)	PROKR2 (17.6%)	CHD7 (7.1%)	FGFR1 (8.5%)	GLI3 (7.5%)
	GLI3 (4.2%)	CHD7 (7.8%)	FGFR1 (13.6%)	PROKR2 (5.2%)	HS6ST1 (1.4%)	CHD7 (9.8%)	TACR3 (7.1%)	IGSF10 (5.4%)	NOTCH1 (7.5%)
	AMN1 (2.1%)	IL17RD (5.9%)	TACR3 (4.5%)	SOX10 (4.3%)	PROKR2 (1.4%)	ANOS1 (7.2%)	PROKR2 (3.6%)	GNRHR (5.4%)	MASTL (7.5%)
	CCKBR (2.1%)	ANOS1 (5.9%)		AXL (3.4%)	TAC3 (1.4%)	ERBB4 (6.5%)	ANOS1 (3.6%)	WDR11 (4.6%)	PROKR2 (5%)
	CRY1 (2.1%)	FGF17 (2%)		GNRHR (3.4%)	FEZF1 (1.4%)	FGFR1 (6.5%)	SOX3 (3.6%)	ANOS1 (4.6%)	AMH (5%)
	CXCR4 (2.1%)	KISS1R (2%)		SEMA3A (2.6%)		EGFR (5.9%)		TACR3 (3.8%)	JAG1 (5%)
	FGF13 (2.1%)	PROK2 (2%)		IL17RD (2.6%)		LHB (5.9%)		PROK2 (3.8%)	IL17RD (5%)
	GAP43 (2.1%)	SEMA3A (2%)		TACR3 (2.6%)		PLXNB1 (0.59%)		DMXL2 (3.1%)	PDE3A (5%)
	GNRH1 (2.1%)	SPRY4 (2%)		ANOS1 (1.7%)		SEMA4D (5.9%)		PROKR2 (2.3%)	ANOS1 (5%)
	GNRHR (2.1%)			FGF8 (1.7%)		EGF (4.6%)		POLR3B (2.3%)	GNRHR (5%)
	IL17RD (2.1%)			HS6ST1 (1.7%)		NRP2 (4.6%)		IL17RD (2.3%)	TAC3 (2.5%)
	JAG1 (2.1%)			WDR11 (1.7%)		B3GNT1 (3.9%)		SPRY4 (1.5%)	TACR3 (2.5%)
	MASTL (2.1%)			GNRH1 (1.7%)		IL17RD (3.9%)		SOX10 (1.5%)	AMHR2 (2.5%)
	NOS1 (2.1%)			KISS1 (1.7%)		NOS1 (3.9%)		SEMA7A (1.5%)	KISS1R (2.5%)
	NOTCH (2.1%)			FGF17 (0.9%)		ROBO3 (3.9%)		SEMA3A (1.5%)	SPRY4 (2.5%)
	NRP2 (2.1%)			PROK2 (0.9%)		DCC (3.3%)		POLR3A (1.5%)	
	PALM2 (2.1%)			KISS1R (0.9%)		MTOR (3.3%)		NSMF (1.5%)	
	PDE3A (2.1%)			TAC3 (0.9%)		SEMA7A (3.3%)		IGFALS (1.5%)	
	PLEHKA5 (2.1%)					DLX5 (2.6%)		GNRH1 (1.5%)	
	RD3 (2.1%)					GNRHR (2.6%)		FGF8 (1.5%)	
	TRAPPC9 (2.1%)					IGF1 (2.6%)		TAC3 (0.8%)	
	TSPAN11 (2.1%)					KISS1R (2.6%)		RNF216 (0.8%)	
						PAX6 (2.6%)		PNPLA6 (0.8%)	
						AXL (2%)		OTX2 (0.8%)	
						CNTN2 (2%)		IGSF1 (0.8%)	
						EBF2 (2%)		FLRT3 (0.8%)	
						EFNA5 (2%)		EBF2 (0.8%)	
						MET (2%)		FGF17 (0.8%)	
						PLXNA1 (2%)			
						SEMA3A (2%)			
						SLIT2 (2%)			
						TACR3 (2%)			
						FEZ1 (1.3%)			
						CCKAR (1.3%)			
						DCAF17 (1.3%)			
						EDNRB (1.3%)			
						EPHA5 (1.3%)			
						GHR (1.3%)			
						HGF (1.3%)			
						NRP1 (1.3%)			
						WDR11 (1.3%)			
						CASR (0.7%)			
						GH1 (0.7%)			
						GNRH1 (0.7%)			
						LEPR (0.7%)			
						LIF (0.7%)			
						NELF(NSMF) (0.7%)			
						PROK2 (0.7%)			
						STS (0.7%)			
						TLE4 (0.7%)			
						TYRO3 (0.7%)			

#### Genetic counseling

Genetic screening is essential in CHH as it can be treated and patients could have a good reproductive prognosis upon treatment (see details in (Boehm et al. 2015; Maione et al. 2018). Genetic counseling should give information on heritability for other family members too, and also required before family planning (Maione et al. 2018).

In certain cases, heritability can be determined relatively easily. For instance, in case of *GNRH1*/*GNRHR*, *TAC3*/*TACR3*, *KISS1*/*KISS1R*, autosomal recessive inheritance pattern is characteristic, while *ANOS1* is inherited as an X-linked trait (Maione et al. 2018). However, for genes of which variants inherited by an autosomal dominant way, the penetrance and expressivity can be variable. In case of *FGFR1* nearly half, regarding *PROK2*/*PROKR2* nearly two-third of the cases exhibit incomplete penetrance and variable expressivity complicating the determination of the inheritance pattern (Maione et al. 2018). Regarding certain genes (e.g. *FGFR1*), de novo mutations are also relatively common that has to be taken into consideration when analyzing pedigrees.

Additionally, together with the availability of NGS, the main challenge is to distinguish true oligogenicity from rare variants which appear as incidental findings and are not related to the phenotype. In determination of oligogenicity, genotype–phenotype co-segregation should be assessed by investigating both the affected and healthy family members. In addition, in diagnosis of oligogenicity, Maione et al. (2018) suggested that oligogenic load has to be correlated with phenotype severity. There are several complicating factors (small families, not available or not compliant family members, incomplete penetrance and variable expressivity) in segregation analysis, still, it is one of the most important way to identify the closest evidence of pathogenicity clinically besides in vitro and in vivo studies (Oliver et al. 2015; Maione et al. 2018). Additionally, in clinical interpretation of variants of unknown significance (VUSs), clinical data (genotype–phenotype segregation) are of utmost important.

Once heritability is assessed, risk of disease transmission can be discussed according to the Mendelian rules.

Prognosis has also to be discussed as approximately 20% of the cases appear to be spontaneously reversible. From genetic point of view, to date *TAC3* and *TACR3* loss of function variants were described to be associated with CHH reversal (Gianetti et al. 2010), but with the increasing data provided by high-throughput NGS platforms, the number of genes connected to this phenomenon will probably increase as well.

## Next-generation sequencing allows evaluation of sequence variants of several genes at the same time in a cost-effective way

Formerly, genetic testing was confined to rare genetic disorders due to their complexity, labour intensity and cost. Now, NGS-based methods are widely available allowing to test even hundreds of genes at the same time. Therefore, NGS has been rapidly integrated into laboratory diagnostics workflows for identification of germline mutations in inherited diseases. Due to its time and cost effectiveness, it is especially useful in cases when several genes have been identified in the background of a certain genetic condition such as CHH.

### NGS-based platform options for clinical genetic diagnostics

Although the technology allows to investigate the sequence of the whole genome (WGS, whole genome sequencing) or exome (WES, whole exome sequencing) currently, the most prevalent applications of NGS in clinical practice are the evaluation of certain genes using targeted gene panels (Di Resta et al. 2018).

As *WGS* covers the whole genome (coding and noncoding regions) it may seem the most preferable choice in identification of pathogenic gene mutations in inherited diseases. The advantage of WGS is that library preparation is straightforward as it does not require target enrichment. Additionally, data obtained from WGS can easily be used for detection of CNVs. However, among NGS approaches it gives the least average depth of coverage and it is still a costly technology (Di Resta et al. 2018). Also, from clinical point of view, the interpretation of noncoding variants and variants of unknown significance (VUSs) make its utility limited.

*WES* aims to cover all coding regions in the genome. Exome contains all of the protein-coding regions of genes and it comprises ~ 1–2% of the genome, yet contains ~ 85% of known disease causing mutations. Its cost is also more preferable and it is a more feasible option comparing to WGS (Di Resta et al. 2018). Usually, the average exome coverage of a WES test is 90–95% due to sequence complexity. WES is sometimes used by clinical laboratories by interpreting only genes which have been already associated with any disease. When mutation has not been identified data analysis can be extended to the remaining exome regions. It has been shown that WES provides diagnosis in approximately of 11–40% of cases where the clinical diagnosis were uncertain (Sawyer et al. 2016). Furthermore, because the depth of coverage for WES is not uniform the sensitivity is usually lower compared to those observed in case of targeted disease panels.

Customized targeted gene panels offer the ability to perform fast and low-cost screening option, therefore, currently, it is the most widely used NGS approach in clinical practice (Di Resta et al. 2018; Wang et al. 2018; Graziola et al. 2019). By focusing on a limited set of genes selected for certain clinical condition, it is able to provide high coverage that increases analytical sensitivity even in detection of mosaicism. Furthermore, because the role of genes included in these panels are known to be associated with the particular condition the detection rate (positive finding) is also higher compared to WES (Di Resta et al. 2018; Wang et al. 2018; Graziola et al. 2019). Targeted panels give the advantage to avoid incidental, secondary findings and to decrease the number of VUSs detected.

Therefore, when the genetic background is well-defined, targeted testing of a gene panel could offer at a relatively low-cost sensitive detection of genetic variants responsible for a disease. However, when no suspect genes stand behind the clinical phenotype, exome sequencing may provide a wider screening option, but in these cases, trio sequencing would allow a more comprehensive result compared to the “only” individual sequencing.

### Workflow of an NGS-based genetic analysis

NGS-based sequencing analysis comprises of three steps: (1) library preparation, (2) parallel sequencing and (3) data analysis and variant interpretation (Oliver et al. 2015).

Molecular genetic analysis is routinely performed using DNA extracted from peripheral blood or buccal mucosa. In our example, we used DNA samples of 38 consecutive patients and 2 family members with hypogonadotropic hypogonadism referred to our diagnostics laboratory. Fourteen patients developed the disease ≤ 18 years [2 girls and 12 boys with an average age of 16.2 year (± 2.1 years)]. Twenty-four patients developed disease in adult age (3 females, 21 males with an average age of 31.8 years (± 12.7 years). Patient characteristics, clinical findings, laboratory results and imaging studies are included in Supplementary Table 1. Our study was approved by the Scientific and Research Committee of the Medical Research Council of Ministry of Health, Hungary (67/PI/2012). All samples were obtained after acquiring written informed consent from all adult patients and permissions were given by parents of all minors. For NGS-based technologies, the amount and quality of input DNA is an essential factor. Degradation or low concentration of DNA may jeopardize the analysis.

For any NGS-based strategy, library preparation is a key step in the laboratory workflow. The instrumentation determines the library preparations, because high-throughput instruments allow larger analysis. Barcodes (unique, short sequences) are used to label different samples enabling pooling patients’ samples into one reaction and decreasing the per-sample cost. Library preparation methods can be grouped into two main categories by principle used for gene amplifications: (1) PCR-based and (2) hybridization-based methods (Butz and Patócs 2019). Although processes using hybridization-based capture are more time consuming and labour intensive, those have the advantage of having greater tolerance against sequence variations (sequence variants and copy-number alteration).

The sequencing characteristics (read length, output read number, cost and run time) of each platform can be different that are needed to be taken into consideration.

For an in-house panel design (gene selection), the recommendation of the European Society of Human Genetics should be followed. Only genes with known relationship between genotype and phenotype should be included in the analysis for diagnostic purposes (Matthijs et al. 2016). Also, the guideline states that “to avoid irresponsible testing, for the benefit of the patients, ‘core disease gene list’ should be established by the clinical and laboratory experts” (Matthijs et al. 2016). Therefore, consensus statements and guidelines, OMIM (Online Mendelian Inheritance in Man) database and literature search should be assessed to assemble genes in a diagnostic panel. For CHH, there is an available European Consensus Statement (Boehm et al. 2015) which was used as a primary guide during our panel design too.

Accordingly, our panel was designed during the first half of 2017. Some CHH-related genes were left out, mostly those which have been already introduced earlier into clinical practice in our laboratory (e.g. genes responsible for combined pituitary deficiency or adrenal diseases) (Halász et al. 2006; Bertalan et al. 2019) or due to the capacity of the applied method. Genes associated with complex syndromes were not present either in our selection owing to our patient profile. Finally, 41 genes were analyzed (see in Supplementary Table 2 and in Table 3).

**Table 3:** CHH gene list and panel performance indicated by coverage (mean read/base ± SD)

Gene name	Ensembl gene ID	Covered region (bp)	Avg coverage/base ± SD
KISS1	ENSG00000170498	594	66 ± 14
RD3	ENSG00000198570	765	77 ± 15
CXCR4	ENSG00000121966	1128	90 ± 23
NRP2	ENSG00000118257	3813	86 ± 18
IL17RD	ENSG00000144730	2997	82 ± 18
GAP43	ENSG00000172020	1062	80 ± 18
GNRHR	ENSG00000109163	1164	79 ± 18
TACR3	ENSG00000169836	1695	76 ± 17
SPRY4	ENSG00000187678	1146	74 ± 12
GLI3	ENSG00000106571	5640	80 ± 15
SEMA3A	ENSG00000075213	3333	64 ± 15
FGF17	ENSG00000158815	948	80 ± 15
GNRH1	ENSG00000147437	456	70 ± 17
FGFR1	ENSG00000077782	3639	83 ± 17
TRAPPC9	ENSG00000167632	5058	75 ± 17
PALM2	ENSG00000243444	1557	79 ± 17
NOTCH1	ENSG00000148400	9705	76 ± 14
NSMF	ENSG00000165802	2421	72 ± 13
MASTL	ENSG00000120539	3354	65 ± 15
FGF8	ENSG00000107831	1092	75 ± 13
CCKBR1	ENSG00000110148	1641	80 ± 15
FSH	ENSG00000131808	567	70 ± 15
PLEKHA5	ENSG00000052126	5082	61 ± 14
PDE3A	ENSG00000172572	4383	69 ± 13
TSPAN11	ENSG00000110900	1239	71 ± 12
AMN1	ENSG00000151743	1194	51 ± 13
AMHR2	ENSG00000135409	2379	83 ± 17
TAC3	ENSG00000166863	723	78 ± 15
DUSP6	ENSG00000139318	1323	74 ± 12
CRY1	ENSG00000008405	2538	64 ± 14
NOS1	ENSG00000089250	6144	87 ± 17
CDH7	ENSG00000081138	3075	72 ± 18
KISS1R	ENSG00000116014	1494	49 ± 19
AMH	ENSG00000104899	1980	42 ± 10
AXL	ENSG00000167601	3882	85 ± 17
LHB	ENSG00000104826	603	32 ± 7
PROKR2	ENSG00000101292	1332	94 ± 20
JAG1	ENSG00000101384	3834	78 ± 16
FLRT3	ENSG00000125848	2067	79 ± 20
ANOS1	ENSG00000011201	2880	41 ± 18
FGF13	ENSG00000129682	1035	39 ± 16

We selected NimbleGene approach to create the appropriate hybridization capture probe set for our gene list using NimbleDesign Software (https://sequencing.roche.com/en/products-solutions/by-category/target-enrichment/software/nimble-design-software.html) targeting the region of interests (exons + /– 30 bp/exon). Capture probe synthesis was done by the supplier. Library was prepared following double capture; the library quantification was performed following the manufacturer’s instructions (NimbleGen SeqCap EZ Library protocol). Sequencing runs were done on Illumina MiSeq instrument using MiSeq Reagent Micro Kit v2.

#### Sequencing data processing, performance analysis

During NGS, huge amount of data is produced which require special bioinformatics handling and analysis (Biesecker and Green 2014), therefore, appropriate hardware, software and expert personnel are required for data analysis (Oliver et al. 2015). Currently, there is no gold standard, freely available tool or filtering settings for bioinformatics analysis related to clinical applications of NGS. Each laboratory has to develop and validate its own pipeline (Oliver et al. 2015).

First step of sequencing data analysis is base calling that is integrated into the instrument’s software. During the next step, raw sequence reads are aligned to the reference human genome (Sayitoğlu 2016). Quality filtering of read alignment defines sensitivity and specificity of the test. Using very strong filtering could lead to loss of variants, while inclusive filters can minimize false negative results but it will increase the burden of confirmatory analysis. Both coverage depth and uniformity are important regarding detection accuracy. In germline testing, a minimum of 20 reads/alleles are required for diagnostic purposes. On the other hand, as read/error ratio increases with the increase of coverage practically 300–500 reads/target has been suggested to be enough for diagnostics (Strom 2016; Deans et al. 2017; Butz and Patócs 2019). Even if the coverage is adequate, it is important to evaluate coverage uniformity in order not to miss certain regions falling below the detection cut-off, because variants not detected will not be further analyzed (Rizzo and Buck 2012). In certain cases, due to sequence complexity, 1–2% of the targeted region may not be covered (Rizzo and Buck 2012).

Variant calling is performed to identify alterations compared to the reference sequence (Oliver et al. 2015). In this step, false sequence variants are omitted by investigating variant allele frequency (VAF) (Lee et al. 2014; Deans et al. 2017). (VAF is the percentage of sequence reads divided by the overall coverage of the particular locus. In germline testing, VAF represents diploid zygosity (near 0 and 100% for homozygosity and near 50% for heterozygosity). Unfortunately, results of different variant calling algorithms do not correlate well, therefore, to maintain technical validity, confirmatory tests are recommended (Trubetskoy et al. 2015; Matthijs et al. 2016; Muller et al. 2016). In germline NGS applications, Sanger sequencing is generally accepted for validation.

As In Vitro Diagnosis (IVD) proved NGS-based assays are not widely available, each laboratory has to develop and validate their own protocols including from sample and library preparation, bioinformatics analysis and quality assurance (Rehm et al. 2013). In our analysis, we followed the Genome Analysis Tool Kit (GATK) Best Practices guideline using the germline short variant discovery (SNPs + Indels) algorithm (DePristo et al. 2011). A minimum coverage of 20 reads was applied as detection filter. In our gene panel, all regions were covered by 71 ± 14 reads/base (see details regarding each gene in Table 3).

The accuracy depends on the depth of sequence coverage therefore NGS gene panels show the highest diagnostic accuracy (Oliver et al. 2015). Indeed, in a recent study, comparing different exome sequencing platforms found that 93.2% of the investigated regions were covered > 10 reads (Kong et al. 2018) (of the covered regions the sensitivity was reported 97.5–99.99%). Comparably, in our panel, 97.2% of the investigated regions (86,329 bp) was covered > 20 read/base. Of the investigated region, 14,532 bp was assessed by Sanger sequencing as well, and all detected variants were identified by both approach, therefore the specificity of our panel was 100%.

#### Variant interpretation

Variant interpretation are guided by expert recommendations for clinical diagnostics [American College of Medical Genetics and Genomics (ACMG), European Society of Human Genetics (ESHG)] which should be followed for all laboratories offering NGS-based diagnostics (Rehm et al. 2013; Richards et al. 2015; Matthijs et al. 2016).

**Fig. 2:**
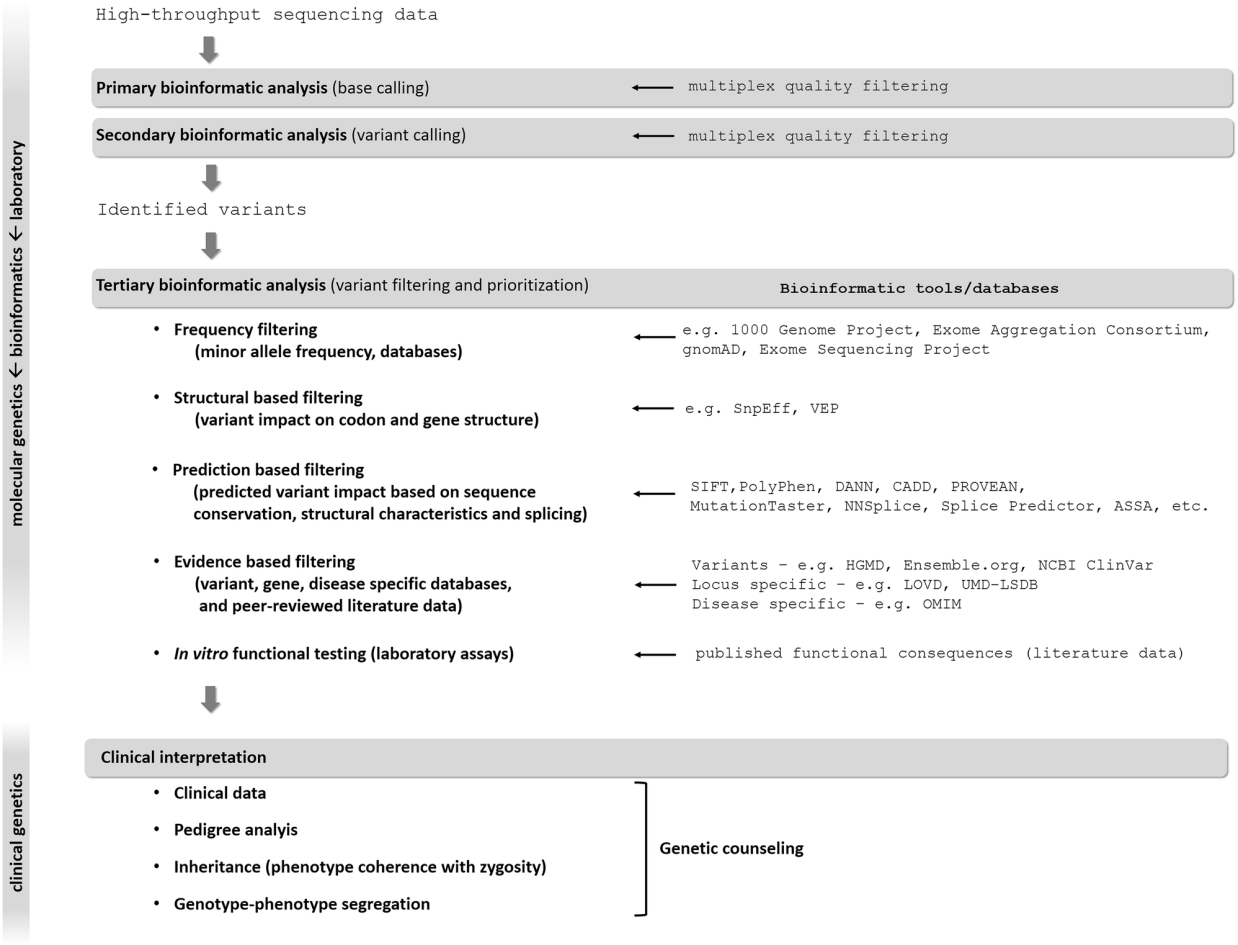
Process of molecular genetic testing by NGS from NGS data analysis to variant interpretation. See details in the text

WGS usually identifies 3–4 million, while WES detects usually 15,000–20,000 variants. Therefore, variant prioritization and interpretation are needed to determine the one or the few pathogenic variants responsible for disease (Fig. 2). First step is to assess the prevalence of certain variants in general population-based databases to filter out frequent variants assuming that pathogenic variants are not common in the broad population. However, in oligogenic diseases, relatively frequent variants can have additional or genetic modifier effect on the phenotype (Maione et al. 2018).

Analyzing the functional consequence of a certain variant may help the interpretation. Using various algorithms (Fig. 2), variants can be classified to have low, moderate or high impact on protein function. It has to be kept in mind that even synonymous variants could sometimes influence splicing and, therefore, amino acid composition of the mature protein resulting in a pathogenic variant (Gianetti et al. 2010; Courage et al. 2019). After classification, further gene, variant and disease specific databases together with peer-reviewed literature data (Fig. 2) can help the accurate interpretation (Richards et al. 2015).

To estimate the pathogenicity of variants of uncertain significance (VUS) is more challenging. Multiple sources of information (variant frequency, in silico predictions of variant effect on protein function and subsidiary functional studies) are needed to be taken into account in order to follow recommendations of guidelines in categorization of a particular variant (Richards et al. 2015; Matthijs et al. 2016). In this framework, in vitro and in vivo functional assays are not always available. These experiments are labour intensive, need longer time, and typically performed as the part of research.

The molecular genetic laboratory report should focus on containing the clinically relevant information for clinicians together with a brief description of all NGS quality metrics (technical characteristics, bioinformatics pipelines, validation), variant annotations and classification (Richards et al. 2015; Matthijs et al. 2016). Disease-specific statements and/or recommendation can greatly guide the interpreter in variant evaluation. The raw data and the full report should also be available upon request.

In our case, for variant filtering, the following parameters were used: minor allele frequency (MAF) cut-off 1%, coding properties (synonymous variants were omitted), and variants’ effects were evaluated by prediction softwares (SNPeffect—Cingolani et al. 2012 and DANN). Variant interpretation was done following the ACMG recommendation (Richards et al. 2015). Additionally, the European Consensus Statement on congenital hypogonadotropic hypogonadism, Human Gene Mutation Database (HGMD) and peer-viewed articles were searched to categorize the detected variants.

All identified Class V, IV and III variants (pathogenic, likely pathogenic and variant of unknown significance, VUS) were validated using conventional bidirectional Sanger sequencing on an Applied Biosystems 3130 Genetic Analyzer System. Following Sanger validation of all pathogenic, likely pathogenic and VUS variants we found 100% of concordance between NGS and Sanger results.

### Pathogenicity of the identified variants, genotype–phenotype correlation

After publication of the CHH expert consensus recommendation 7 NGS panel studies have been published about the molecular genetic analysis of CHH. Including our current study, a total of 588 patients with CHH and 72 patients with CDGP were evaluated. Using various NGS approaches of these patients 262 (44%) with CHH and 5 (5.5%) with CDGP diagnosis carried a pathogenic variant (Table 2).

Regarding phenotype–genotype correlation some authors reported inconclusive results and little co-segregation by analyzing pedigrees in their cohorts (Aoyama et al. 2017; Zhou et al. 2018), probably due to the complex genetic background of CHH. However, differences in genetic profile among populations are indicated in Chinese and Japanese cohorts (Aoyama et al. 2017; Zhou et al. 2018). Zhou et al. reported that in Chinese population, cryptorchidism was the most common accompanying feature in addition to CHH, but no single gene in their panel showed association with this abnormality (Zhou et al. 2018). Wang et al. reported that the frequency of *PROKR2* mutations was higher in dual CHH patients (showing hypothalamic and/or pituitary defects with testicular hypoplasia) when compared to other CHH cases. The authors suggested that testicular development are affected in early life reflecting the results of animal experiments where the loss of *Prokr2* compromised the integrity of the testicular vasculature (Wang et al. 2017).

In Kallmann syndrome, anosmia/hyposmia is part of the clinical picture, and *ANOS1, CHD7, FGFR1, PROK2, PROKR2,* and *SEMA3A* variants were reported to be involved in isolated congenital anosmia (Alkelai et al. 2017). The genetic background of CHH reversal is still unclear, however, the recently identified *IGSF10* and *GNRHR* variants in addition to previously reported *TAC3* and *TACR3* variants need further studies for clarification of their pathogenic role (Amato et al. 2019).

The genetic background of CDGP and CHH share common aspects, they also have distinct profiles. In CHH patients, both mutations and oligogenicity of CHH genes have been more commonly identified compared to CDGP (Cassatella et al. 2018). In turn, the genetic profile of CDGP resembled more closely to those founded in control cohort. No pathogenic alterations, but frequent (MAF 1.0–2.5%) genetic variants have been more commonly detected in CDGP compared to controls suggesting their genetic modifier’s role (Cassatella et al. 2018).

In CHH, oligogenicity was reported between 0 and 19% (Table 2). Interestingly, in Japanese populations Aoyama et al. did not find any patients with CHH caused by di-/oligogenic mutations (Aoyama et al. 2017) while Quaynor et al. described that the majority of the suggested di- and oligogenic background could be supported by pedigree analysis (9/11 pedigrees) in their cohort (Quaynor et al. 2016). Nevertheless, others suggested that with the increase of the numbers of genes investigated the detection rate of oligogenicity will increase (Amato et al. 2019) making difficult to prove the true role of di/oligogenic findings (Maione et al. 2018).

Our panel identified a total of 31 variants in 22 probands (1)13 patients with only 1 variant per individual; (2) 1 patient with compound heterozygous variants in the *GNRHR* gene; (3) 8 patients with 2 heterozygous variants in two different genes (digenic case). The digenic rate was 21% (8/38). The eight pathogenic/likely pathogenic variants, detected in eight patients, hence the genetic cause was clearly identified in 21% of cases tested. (8/38 = 21%). In three patients the pathogenic mutations were detected together with variants of unknown significance (VUSs) (Patient IDs: 10, 29 and 31). In addition, in 14 patients, only VUSs were identified (in 6 patients, 2 and in 8 patients, 1 VUS) which need further studies (Table 4). Grouping our patients into adult and pediatric groups, our data show that clearly pathogenic variants in adult patients were identified in 7 (29%; 7/24), while in 1 pediatric cases (7%; 1/14).

**Table 4:** Variants identified by targeted NGS panel in 38 CHH probands

Patient ID	Sex	Clinical diagnosis	Gene_name	Inheritance	Variant identified			ACMG classification	Minor allele frequency (MAF)		Prediction (DANN)	Gene Reference	Variant Reference (HGMD ID, Ref)
					HGVS cdna	HGVS prot	Zygosity		Total	Eu (non-Finnish)			
Probands (*n* = 38)													
3	F	KS, anosmia (primer amenorrhea)	PROKR2	AD, contributes to oligogenicity	NM_144773.3:c.518 T > G	p.Leu173Arg	Heterozygous	Likely pathogenic	0.00229	0.00347	0.9976	Boehm et al. (2015)	Dodé et al. (2006), Cole et al. (2008), Monnier et al. (2009), Abreu et al. (2010), Reynaud et al. (2012), Cassatella et al. (2018), Amato et al. (2019)
5	M	KS	AMH	AR (Malone et al. 2019)	NM_000479.4:c.1556C > T	p.Ala519Val	Heterozygous	VUS	0.00159	0.00252	0.9582	Malone et al. (2019)	Novel in IHH (It was deteceted in a DSD case by Hughes et al. (2019))
			JAG1	AD (Quaynor et al. 2016)	NM_000214.3:c.3109G > A	p.Asp1037Asn	Heterozygous	VUS	0.00000795	0.0000176	0.9918	Quaynor et al. (2016)	Novel in IHH
10	M	KS, anosmia, pubertal delay	FGFR1	AD, contributes to oligogenicity	NM_001174067.1:c.417delC^a^	p.Ser140ArgfsTer43	heterozygous	Pathogenic	Absent	Absent	na	Boehm et al. (2015)	Novel in IHH
			IL17RD	AD, AR or digenic dominant, contributes to oligogenicity	NM_017563.5:c.1696C > T	p.Pro566Ser	Heterozygous	VUS	0.0144	0.0214	0.9981	Boehm et al. (2015)	Novel in IHH [Amato et al. (2019) found a pathogenic variant in the same codon: c.1697C > T; p.Pro566Leu]
12	M		PDE3A	Digenic (Quaynor et al. 2016)	NM_000921.4:c.293C > A	p.Ala98Glu	Heterozygous	VUS	0.000431	0.000884	0.7627	Quaynor et al. (2016)	Novel in IHH
13	M	KS	ANOS1	XR, contributes to oligogenicity	NM_000216.4:c.1700G > A	p.Gly567Asp	Hemizygous	Likely pathogenic	Absent	Absent	0.9959	Boehm et al. (2015)	Novel in IHH
15	M	KS, anosmia, parosmia	GNRHR	AR, contributes to oligogenicity	NM_000406.2:c.416G > A	p.Arg139His	Heterozygous	Likely pathogenic	0.000144	0.000168	0.9994	Boehm et al. (2015)	Costa et al. (2001), Topaloglu et al. (2006), Beneduzzi et al. (2014)
16	M	KS	GLI3	digenic (Quaynor et al. 2016)	NM_000168.6:c.2179G > A	p.Gly727Arg	Heterozygous	VUS	0.00527	0.00755	0.9994	Quaynor et al. (2016)	Novel in IHH (Radhakrishna et al. 1999 detected in postaxial polydactyly type-A/B)
			JAG1	AD (Quaynor et al. 2016)	NM_000214.3:c.3320C > T	p.Ser1107Phe	Heterozygous	VUS	Absent	Absent	0.9929	Quaynor et al. (2016)	Novel in IHH
17	M	KS	NOTCH1	AD? (Quaynor et al. 2016)	NM_017617.5:c.4049G > T	p.Arg1350Leu	Heterozygous	VUS	0.000604	0.00103	0.6583	Quaynor et al. (2016)	Novel in IHH
18	M	nCHH, pubertal delay	GLI3	digenic (Quaynor et al. 2016)	NM_000168.6:c.2179G > A	p.Gly727Arg	Heterozygous	VUS	0.00527	0.00755	0.9994	Quaynor et al. 2016	Novel in IHH [Radhakrishna et al. (1999) detected in postaxial polydactyly type-A/B]
20	M	KS, anosmia, pubertal delay	NOTCH1	AD? (Quaynor et al. 2016)	NM_017617.5:c.3860G > A	p.Arg1287His	Heterozygous	VUS	0.0000688	0.000191	0.9977	Quaynor et al. (2016)	Novel in IHH
23	M	KS, anosmia	FGFR1	AD, contributes to oligogenicity	NM_001174067.1:c.1012delT^a^	p.Tyr338MetfsTer5	Heterozygous	Pathogenic	*–*	*–*	na	Boehm et al. (2015)	Novel in IHH
26	M	nCHH, pubertal delay	MASTL	AD, di-, trigenic (Quaynor et al. 2016)	NM_001172303.2:c.2120A > T	p.His707Leu	Heterozygous	VUS	0.000004	0.000000	0.8767	Quaynor et al. (2016)	Novel in IHH
			TAC3	AR, contributes to oligogenicity	NM_013251.4:c.248A>C	p.His83Pro	heterozygous	VUS	0.000008	0.000009	0.977	Boehm et al. (2015)	Novel in IHH [Cassatella et al. (2018) found a pathogenic variant in the same codon: c.248A>G; p.His83Arg]
29	M	nCHH	GNRHR	AR, contributes to oligogenicity	NM_000406.2:c.350 T > G	p.Leu117Arg	Heterozygous	Likely pathogenic	absent	absent	0.9971	Boehm et al. (2015)	HGMD ID: CM128135 Gürbüz et al. (2012), Cassatella et al. (2018)
			GNRHR	AR, contributes to oligogenicity	NM_000406.2:c.158A > T	p.Asn53Ile	Heterozygous	VUS	Absent	Absent	0.9881	Boehm et al. (2015)	Novel in IHH
30	M	nCHH	NOTCH1	AD? (Quaynor et al. 2016)	NM_017617.5:c.2734C > T	p.Arg912Trp	Heterozygous	VUS	0.001710	0.002570	0.9989	Quaynor et al. (2016)	Novel in IHH [Digilio et al. (2019) reported in association with cardiovascular anomalies]
			PROKR2	AD, contributes to oligogenicity	NM_144773.3:c.701G > A	p.Gly234Asp	Heterozygous	VUS	0.000008	0.000018	0.9981	Boehm et al. (2015)	HGMD ID: CM1211225 Tommiska et al. (2013)
31	M	KS, hyposmia	ANOS1	XR, contributes to oligogenicity	NM_000216.4:c.1246dupA^a^	p.Thr416AsnfsTer29	Hemizygous	Pathogenic	*–*	*–*	*na*	Boehm et al. (2015)	Novel in IHH
			MASTL	AD, di-, trigenic (Quaynor et al. 2016)	NM_001172303.2:c.871C > T	p.Leu291Phe	Heterozygous	VUS	0.000540	0.000940	0.9989	Quaynor et al. (2016)	Novel in IHH
32	M	nCHH, pubertal delay	TACR3	AR, contributes to oligogenicity	NM_001059.2:c.1345G>T	p.Ala449Ser	Heterozygous	VUS	0.000162	0.000305	0.9737	Boehm et al. (2015)	Tusset et al (2012) reported in a case of CDGP
33	M	nCHH, pubertal delay	AMHR2	AR (Malone et al. 2019)	NM_020547.3:c.1484G > A	p.Arg495Gln	Heterozygous	Likely pathogenic^a^	0.000203	0.000009	0.9995	Malone et al. (2019)	Novel in IHH
35	M	pubertal delay	KISS1R	AR	NM_032551.5:c.581C > A	p.Ala194Asp	Heterozygous	VUS	0.000352	0.000882	0.977	Boehm et al. (2015)	Miraoui et al. (2013)
36	M	pubertal delay	MASTL	AD, di-, trigenic (Quaynor et al. 2016)	NM_001172303.2:c.1505A > G	p.Asp502Gly	Heterozygous	VUS	Absent	Absent	0.9219	Quaynor et al. (2016)	Novel in IHH
37	M	pubertal delay	SPRY4	AD	NM_030964.3:c.626G > A	p.Cys209Tyr	Heterozygous	VUS^**b**^	0.002170	0.003450	0.9971	Miraoui et al. (2013)	HGMD ID: CM133832 Miraoui et al. (2013)
			AMH	AR (Malone et al. 2019)	NM_000479.4:c.761G > C	p.Arg254Pro	Heterozygous	VUS^**b**^	0.000015	0.000000	0.9626	Malone et al. (2019)	Novel in IHH
39	M	KS	FGFR1	AD, contributes to oligogenicity	NM_001174067.1:c.367G>T	p.Asp123Tyr	Heterozygous	VUS	0.000059	0.000000	0.9944	Boehm et al. (2015)	Novel in IHH (It was reported in deafness variation database https://deafnessvariationdatabase.org/)
			GLI3	digenic (Quaynor et al. 2016)	NM_000168.6:c.1163C > T	p.Pro388Leu	Heterozygous	VUS	Absent	Absent	0.7657	Quaynor et al. (2016)	Novel in IHH
40	M	KS	FGFR1	AD, contributes to oligogenicity	NM_001174067.1:c.550C > A	p.Pro184Thr	Heterozygous	VUS	Absent	Absent	0.9974	Boehm et al. (2015)	Novel in IHH

**Gene references:**

Boehm et al. (2015), Malone et al. (2019), Quaynor et al. (2016)

**Variant references:**

Dodé et al. (2006), Cole et al. (2008), Monnier et al. (2009), Abreu et al. (2010), Reynaud et al. (2012), Cassatella et al. (2018), Amato et al. (2019), Hughes et al. (2019), Costa et al. (2001), Topaloglu et al. (2006), Beneduzzi et al. (2014), Radhakrishna et al. (1999), Gürbüz et al. (2012), Digilio et al. (2019), Tommiska et al. (2013), Tusset et al. (2012), Miraoui et al. (2013)

^a^After family screening, we considered this variant not disease causing as the unaffected father carried the same variant in heterozygous form

^b^After family screening, we considered these variants not disease causing as the unaffected father carried both variants in heterozygous form

In pediatric cases where healthy parents were available for genetic test, we performed family screening. Healthy parents of patients with IDs “26”, “33” and “37” were available for assessment of variant pathogenicity. By sequencing the particular variants in unaffected parents, we concluded that combination of *SPRY4* p.Cys209Tyr and *AMH* p.Arg254Pro variants were probably not disease causing as the healthy father also carried the same genotype. Our family screening suggests that the originally predicted as likely pathogenic variant (*AMHR2* p.Arg495Gln) is benign for CHH and might be VUS for delayed puberty because the unaffected father carried the same variant in heterozygous form. The co-existence of *TAC3* p.His83Pro with *MASTL* p.His707Leu (Patient “26”) could be potentially pathogenic as they were inherited from different parents. Naturally, in CDGP cases, follow-up time (onset of puberty could spontaneously occur) and further studies will possibly clarify the pathogenicity of these variants.

During the family screening of our case, Patient ID “9” the same pathogenic mutation was identified in his clinically affected brother (Patient ID “10”). There are phenotypes are similar, however, in Patient ID “10” anosmia was present.

It is noteworthy that some alterations (such as repetitive sequences, copy-number variations, long insertion–deletions, structural variants, aneuploidy or epigenetic alterations) are not well detectable by NGS methods Therefore, when these types of alterations are expected the appropriate method (such as multiplex ligation probe amplification (MLPA) or microarray-based comparative genomic hybridization (aCGH)) should be used.

## Summary and conclusion

Congenital hypogonadotropic hypogonadism has a heterogeneous clinical phenotype and genetic background. Especially in pediatric cases, even clinical diagnosis can be challenging (ie. pubertal delay vs. hypogonadism). Genetics data regarding hypogonadotropic hypogonadism with the wider availability of next-generation sequencing are increasing but appropriate tool and expertise are needed for correct interpretation of these results in clinical practice. Based on recent data, in more 50% of cases, the disease causing genetic alterations could be found. In house developed gene panels together with appropriate validation steps have at least the same diagnostic accuracy as the WES. The main challenge in NGS-based methods is the interpretation of variants with unknown significance. For clinical point of view, a great majority of data generated by exome and panel sequencing have still been waiting for clinical validation. The potentially new candidate genes and variants have to be further analyzed functionally (in vitro and in vivo animal experiments) together with thorough clinical genotype–phenotype investigations to prove their disease causing effects. The latter is especially challenging in CHH as the clinical phenotype cover a broad spectrum even in cases harboring the same mutation. In CHH, another great challenge is to distinguish true oligogenic inheritance from incidental, rare findings that are not in relation with CHH. The difficulty in determining inheritance due to non-complete penetrance and variable expressivity together with oligogenicity could mean a difficult situation for genetic counselors. However, over time with the increasing genetic data linked to clinical information will reveal the complex genetic landscape of CHH and eventually it will help variant interpretation.

## Electronic supplementary material

Below is the link to the electronic supplementary material.

Supplementary file1 (DOCX 17 kb)

Supplementary file2 (DOCX 38 kb)
