# Supplementary material for: Molecular genetic diagnostics of hypogonadotropic hypogonadism: from panel design towards result interpretation in clinical practice
Source: Hum Genet. 2020 Mar 28;140(1):113–34. doi: 10.1007/s00439-020-02148-0 (PMC7864839; doi:10.1007/s00439-020-02148-0)
Supplement: Supplementary file 1 — Supplementary file1 (DOCX 17 kb) [file 439_2020_2148_MOESM1_ESM.docx]

**Supplementary Table 1.** CHH patient characteristics.

Patient nr ”9” and “10” were brothers and patient “11” and “12” were father and son.

KS: Kallmann syndrome, nCHH: normosmic congenital hypogonadotropic hypogonadism, HH: hypogonadotropic hypogomadism, FSH: follicle-stimulating hormone, LH: luteinizing hormone, E2: estradiol, T: testosterone

| **Patient ID** | **Sex** | **Age** | **Referring clinical diagnosis** | **Hormone profile** | **Brain MRI** | **Phenotypes** |
| --- | --- | --- | --- | --- | --- | --- |
| 1 | M | 23 | KS |  | - | nCHH |
| 2 | M | 23 | KS |  | - | nCHH |
| 3 | F | 30 | KS | LH: 0.18 mU/mL → 3.45 mU/mL;  FSH: 0.79 mU/mL → 6.11 mU/mL;  E2: 6,4 pg/mL | normal | KS (primary amenorrhea, anosmia), alopecia |
| 4 | F | 40 | KS | LH: 0.1;  FSH: 0.1;  E2: 71 pg/mL | normal | nCHH, primary amenorrhea, uterus hypoplasia, small ovaries |
| 5 | M | 42 | KS |  | - | nCHH |
| 6 | M | 23 | KS | LH: 1.2 mU/mL;  FSH: 0.3 mU/mL;  T: 1.1 nmol/L | normal | KS (HH, anosmia) |
| 7 | M | 32 | KS | LH <0.1 mU/mL;  FSH: <0.1 mU/mL;  T: 2.59 nmol/L | - | KS (HH, criptorchism, delayed puberty, anosmia) |
| 8 | M | 29 | KS | - | - | nCHH |
| 9 | M | 21 | Pubertal delay | LH: 0.8 mU/L ;  FSH: 1.2 mU/L ;  T: 0.81 ng/ml | - | nCHH, pubertal delay |
| 10 | M | 25 | KS | LH: 0.2 mU/L;  FSH: 1 mU/L;  T < 0,43 ng/mL | - | KS (HH, pubertal delay, anosmia) |
| 11 | M | 61 | KS | - | - | - |
| 12 | M | 18 | KS | - | - | - |
| 13 | M | 39 | KS | - | - | nCHH |
| 14 | M | 40 | KS | - | - | KS (HH, anosmia, parosmia) |
| 15 | M | 30 | KS | - | - | - |
| 16 | M | 39 | KS | - | - | - |
| 17 | M | 29 | KS | - | - | criptorchism |
| 18 | M | 19 | Pubertal delay | - | - | nHH, pubertal delay |
| 19 | F | 16 | Pubertal delay | LH < 0.07 → 0.55 mU/mL;  FSH: 0.3 → 2.46 mU/mL;  E2 <43.6 pmol/l | normal | nCHH, pubertas tarda, primary amenorrhea |
| 20 | M | 18 | KS, Pubertal delay | - | Rectus gyrus and orbital medial gyrus are not separable, olfactory sulcus is missing | KS (HH, pubertal delay, anosmia) |
| 21 | M | 19 | Pubertal delay | - | - | - |
| 22 | M | 72 | CHH | T: 1.81 ng/mL | - | nHH |
| 23 | M | 21 | KS | LH: 0.73 → 8.8 IU/l;  FSH: 1.59 → 6.2 IU/l;  T: 0,460 nmol/l | normal | KS (HH, anosmia) |
| 24 | M | 15 | nCHH | FSH: 2.08 IU/L;  LH: 0.34 IU/L ;  T: 0.27 nmol/L | normal | nCHH |
| 25 | F | 21 | KS | FSH: 0.40 IU/L;  LH: 0.05 IU/L | right hypoplastic, left absent olfactory bulb | KS (HH, anosmia) |
| 26 | M | 16 | nCHH, Pubertal delay | FSH: 3.84 IU/L,  LH: 0.37 IU/L;  T: 0.13 nmol/L | normal | nCHH, pubertal delay |
| 27 | M | 16 | nCHH | - | normal | nHH |
| 28 | F | 18 | nCHH | LH < 0.1;  FSH: 0.8 | normal | nHH, primary amenorrhea |
| 29 | M | 27 | nCHH | LH < 0.1;  FSH: 0.2;  T: 0.39 nmol/L | - | nCHH |
| 30 | M | 11 | nCHH | LH: < 0.1;  FSH: 0.37;  T <0.087 nmol/L | - | nCHH |
| 31 | M | 19 | KS | LH: 0.14;  FSH: 0.89;  T <0.64 pg/mL | - | micropenis, orchidopexia, , hyposmia, left kidney agenesia |
| 32 | M | 13 | nCHH, Pubertal delay | LH: <0.1 → 0.2 IU/l;  FSH: <0.3 → 1.1 IU/l;  T: 0.460 nmol/l | - | nCHH, pubertal delay |
| 33 | M | 18 | nCHH, Pubertal delay | LH: 0.41;  FSH: 3.82;  T <0.15 ng/mL | normal | nHH, pubertal delay, short stature, M. Hodgkin |
| 34 | M | 20 | Pubertal delay | LH <2.18 → 7.8 IU/l;  FSH: 4.09 → 5.91 IU/l;  T: 0.25 ng/mL | small adenohypophysis | |
| 35 | M | 18 | Pubertal delay | LH <0.92 → 7.75 IU/l;  FSH <4.04 → 4.89 IU/l;  T: 0.16 ng/mL | - | nCHH, pubertal delay |
| 36 | M | 17 | Pubertal delay | LH <0.28 → 4.73 IU/l;  FSH: 1.35 → 3.05 IU/l;  T 0.93 ng/mL | - | nCHH, pubertal delay, short stature |
| 37 | M | 17 | Pubertal delay | LH <0.02 → 1.98 IU/l;  FSH: 0.87 → 4.16 IU/l;  T 0.2 ng/mL | normal | nCHH, pubertal delay |
| 38 | M | 17 | Pubertal delay | LH <0.71 → 11.62 IU/l;  FSH: 2.47 → 4.9 IU/l;  T: 0.13 ng/mL | - | nCHH, pubertal delay |
| 39 | M | 36 | KS | LH: 0.7;  FSH: 0.8;  T <0.84 ng/mL | - | nCHH |
| 40 | M | 58 | KS | LH <0.01;  FSH <0.05;  T <1.85 ng/mL | - | nCHH |
