# Supplementary material for: Molecular genetic diagnostics of hypogonadotropic hypogonadism: from panel design towards result interpretation in clinical practice
Source: Hum Genet. 2020 Mar 28;140(1):113–34. doi: 10.1007/s00439-020-02148-0 (PMC7864839; doi:10.1007/s00439-020-02148-0)
Supplement: Supplementary file 2 — Supplementary file2 (DOCX 38 kb) [file 439_2020_2148_MOESM2_ESM.docx]

**Supplementary Table 2.** CHH gene panel composition of recently published high-throughput NGS panel studies compared to expert consensus list (Boehm et al. 2015)

|  | Boehm et al. 2016 - European consensus statement: 31 genes are discussed | Quayhor et al. 2016 | Wang et al. 2017 | Aoyama et al. 2017 | Cassatella et al. 2018 | Zhou et al. 2018 | Kim et al. 2018 | Amato et al. 2019 | Current study |
| --- | --- | --- | --- | --- | --- | --- | --- | --- | --- |
| Nr of genes investigated: | | 261 | 164 | 27 | 25 | 83 | 69 | 36 | 43 |
| Gene name: |  |  |  |  |  |  |  |  |  |
| ACSM4 |  | X | Gene list is not specificated |  |  |  |  |  |  |
| AEBP2 |  | X |  |  |  |  |  |  |  |
| AKAP1 |  | X |  |  |  |  |  |  |  |
| AKAP2 |  | X |  |  |  |  |  |  |  |
| AKAP3 |  | X |  |  |  |  |  |  |  |
| AKAP4 |  | X |  |  |  |  |  |  |  |
| ALDH1A1 |  | X |  |  |  |  |  |  |  |
| ALDH1A2 |  | X |  |  |  |  |  |  |  |
| AMH |  |  |  |  |  |  |  |  | X |
| AMHR2 |  |  |  |  |  |  |  |  | X |
| AMN1 |  | X |  |  |  |  |  |  | X |
| ANKRD26 |  | X |  |  |  |  |  |  |  |
| ANOS1 (KAL1) | X | X |  | X | X | X | X | X | X |
| ARL6 (BBS3) |  |  |  |  |  |  | X |  |  |
| ARNTL2 |  | X |  |  |  |  |  |  |  |
| ASUN |  | X |  |  |  |  |  |  |  |
| ATE1 |  | X |  |  |  |  |  |  |  |
| AXL | X | X |  |  | X | X | X |  | X |
| B3GNT1 |  |  |  |  |  | X |  |  |  |
| BBS1 |  |  |  |  |  |  | X |  |  |
| BBS10 |  |  |  |  |  |  | X |  |  |
| BBS12 |  |  |  |  |  |  | X |  |  |
| BBS2 |  |  |  |  |  |  | X |  |  |
| BBS4 |  |  |  |  |  |  | X |  |  |
| BBS5 |  |  |  |  |  |  | X |  |  |
| BBS7 |  |  |  |  |  |  | X |  |  |
| BBS9 |  |  |  |  |  |  | X |  |  |
| BDNF |  | X |  |  |  |  |  |  |  |
| BMP2 |  | X |  |  |  |  |  |  |  |
| BMP4 |  | X |  |  |  |  |  |  |  |
| BMP7 |  | X |  |  |  |  |  |  |  |
| BPIFB4 |  | X |  |  |  |  |  |  |  |
| BRN2 |  | X |  |  |  |  |  |  |  |
| C12ORF70 |  | X |  |  |  |  |  |  |  |
| C12ORF71 |  | X |  |  |  |  |  |  |  |
| CACNA1B |  | X |  |  |  |  |  |  |  |
| CADM2 |  | X |  |  |  |  |  |  |  |
| CAPRIN2 |  | X |  |  |  |  |  |  |  |
| CAS |  | X |  |  |  |  |  |  |  |
| CCDC141 |  | X |  |  |  |  |  |  |  |
| CASR |  |  |  |  |  | X |  |  |  |
| CCDC91 |  | X |  |  |  |  |  |  |  |
| CCK |  |  |  |  |  | X |  |  |  |
| CCKAR |  | X |  |  |  | X |  |  |  |
| CCKBR1 |  | X |  |  |  |  |  |  | X |
| CGA |  | X |  |  |  |  |  |  |  |
| CHAT |  | X |  |  |  |  |  |  |  |
| CHD7 | X | X |  | X | X | X | X | X | X |
| CMAS |  | X |  |  |  |  |  |  |  |
| CNTN2 |  | X |  |  |  | X |  |  |  |
| CPE |  |  |  |  |  |  | X |  |  |
| CRY1 |  | X |  |  |  |  |  |  | X |
| CRY2 |  | X |  |  |  |  |  |  |  |
| CTXN3 |  | X |  |  |  |  |  |  |  |
| CXCL12 |  | X |  |  |  | X |  |  |  |
| CXCR4 |  | X |  |  |  | X |  |  | X |
| DCAF17 |  |  |  |  |  | X | X |  |  |
| DCC |  | X |  |  |  | X |  |  |  |
| DHH |  | X |  |  |  |  |  |  |  |
| DLX1 |  | X |  |  |  |  |  |  |  |
| DLX2 |  | X |  |  |  |  |  |  |  |
| DLX5 |  | X |  |  |  | X |  |  |  |
| DMXL2 | X |  |  |  |  |  | X | X |  |
| DUSP6 |  |  |  | X |  | X | X | X | X |
| EBF2 |  | X |  |  |  | X |  | X |  |
| EDN1 |  |  |  |  |  | X |  |  |  |
| EDNRB |  |  |  |  |  | X |  |  |  |
| EFNA5 |  | X |  |  |  | X |  |  |  |
| EGF |  |  |  |  |  | X |  |  |  |
| EGFR |  | X |  |  |  | X |  |  |  |
| EGR1 |  | X |  |  |  |  |  |  |  |
| EMX1 |  | X |  |  |  | X |  |  |  |
| EMX2 |  | X |  |  |  | X |  |  |  |
| EPHA5 |  |  |  |  |  | X |  |  |  |
| ERGIC2 |  | X |  |  |  |  |  |  |  |
| ERBB4 |  |  |  |  |  | X |  |  |  |
| ETNK1 |  | X |  |  |  |  |  |  |  |
| FAM60A |  | X |  |  |  |  |  |  |  |
| FAR2 |  | X |  |  |  |  |  |  |  |
| FEZF1 | X |  |  |  | X | X | X |  |  |
| FGF1 |  | X |  |  |  |  |  |  |  |
| FGF10 |  | X |  |  |  |  |  |  |  |
| FGF11 |  | X |  |  |  |  |  |  |  |
| FGF12 |  | X |  |  |  |  |  |  |  |
| FGF13 |  | X |  |  |  |  |  |  | X |
| FGF14 |  | X |  |  |  |  |  |  |  |
| FGF16 |  | X |  |  |  |  |  |  |  |
| FGF17 | X | X |  | X | X | X | X | X | X |
| FGF18 |  | X |  |  |  | X |  |  |  |
| FGF19 |  | X |  |  |  |  |  |  |  |
| FGF2 |  | X |  |  |  |  |  |  |  |
| FGF20 |  | X |  |  |  |  |  |  |  |
| FGF21 |  | X |  |  |  |  |  |  |  |
| FGF22 |  | X |  |  |  |  |  |  |  |
| FGF3 |  | X |  |  |  |  |  |  |  |
| FGF4 |  | X |  |  |  |  |  |  |  |
| FGF5 |  | X |  |  |  |  |  |  |  |
| FGF6 |  | X |  |  |  |  |  |  |  |
| FGF7 |  | X |  |  |  |  |  |  |  |
| FGF8 | X | X |  | X | X | X | X | X | X |
| FGF9 |  | X |  |  |  |  |  |  |  |
| FGFR1 | X | X |  | X | X | X | X | X | X |
| FGFR1OP2 |  | X |  |  |  |  |  |  |  |
| FGFR2 |  | X |  |  |  |  |  |  |  |
| FGFR3 |  | X |  |  |  |  |  |  |  |
| FLRT3 |  |  |  | X |  | X | X | X | X |
| FOXH1 |  | X |  |  |  |  |  |  |  |
| FSHB |  | X |  | X |  | X | X |  | X |
| FSTL5 |  | X |  |  |  |  |  |  |  |
| GABABR1 |  | X |  |  |  |  |  |  |  |
| GADL1 |  | X |  |  |  |  |  |  |  |
| GAL |  | X |  |  |  |  |  |  |  |
| GALR1 |  | X |  |  |  |  |  |  |  |
| GALR2 |  | X |  |  |  |  |  |  |  |
| GALR3 |  | X |  |  |  |  |  |  |  |
| GAP43 |  | X |  |  |  |  |  |  | X |
| GAS6 |  | X |  |  |  | X |  |  |  |
| GATA2 |  | X |  |  |  |  |  |  |  |
| GATA3 |  | X |  |  |  |  |  |  |  |
| GATA4 |  | X |  |  |  |  |  |  |  |
| GDF1 |  | X |  |  |  |  |  |  |  |
| GDNF |  | X |  |  |  |  |  |  |  |
| GH1 |  |  |  |  |  | X |  |  |  |
| GHR |  |  |  |  |  | X |  |  |  |
| GHSR |  |  |  |  |  |  |  | X |  |
| GLI1 |  | X |  |  |  |  |  |  |  |
| GLI2 |  | X |  |  |  |  |  |  |  |
| GLI3 |  | X |  |  |  |  |  |  | X |
| GNRH1 | X | X |  | X | X | X | X | X | X |
| GNRHR | X | X |  | X | X | X | X | X | X |
| GPR54 |  |  |  |  |  |  |  |  |  |
| GPX6 |  | X |  |  |  |  |  |  |  |
| GRG |  | X |  |  |  |  |  |  |  |
| GRG4 |  | X |  |  |  |  |  |  |  |
| GRG5 |  | X |  |  |  |  |  |  |  |
| GRIN1 |  | X |  |  |  |  |  |  |  |
| GRIN2 |  | X |  |  |  |  |  |  |  |
| GSTM1 |  | X |  |  |  |  |  |  |  |
| H3F3C |  | X |  |  |  |  |  |  |  |
| HCRT |  | X |  |  |  |  |  |  |  |
| HCRTR1 |  | X |  |  |  |  |  |  |  |
| HDAC8 |  |  |  |  |  |  | X |  |  |
| HESX1 | X | X |  |  |  | X | X |  |  |
| HFE |  |  |  |  |  |  | X |  |  |
| HGF |  | X |  |  |  | X |  |  |  |
| HS6ST1 | X | X |  | X | X | X | X | X |  |
| IFT57 |  | X |  |  |  |  |  |  |  |
| IGF1 |  | X |  |  |  | X |  |  |  |
| IGF1R |  | X |  |  |  |  |  |  |  |
| IGFALS |  |  |  |  |  |  |  | X |  |
| IGSF1 |  |  |  |  |  |  |  | X |  |
| IGSF10 |  |  |  |  | X |  |  | X |  |
| IL17RD (SEF) |  | X |  | X | X | X | X | X | X |
| IPO8 |  | X |  |  |  |  |  |  |  |
| IRF2BPL (EAP1) |  |  |  |  |  |  | X |  |  |
| ITGB1 |  |  |  |  |  | X |  |  |  |
| JAG1 |  | X |  |  |  |  |  |  | X |
| KCNK9 |  | X |  |  |  |  |  |  |  |
| KCTD11 |  | X |  |  |  |  |  |  |  |
| KIAA0528 |  | X |  |  |  |  |  |  |  |
| KISS1 | X | X |  | X | X | X | X | X | X |
| KISS1R | X | X |  | X | X | X | X | X | X |
| KLF6 |  | X |  |  |  |  |  |  |  |
| KLF7 |  |  |  |  |  | X |  |  |  |
| KLHDC5 |  | X |  |  |  |  |  |  |  |
| LEP | X | X |  | X | X | X | X |  |  |
| LEPR | X | X |  | X | X | X | X |  |  |
| LHB |  | X |  | X |  | X | X |  | X |
| LHX3 |  | X |  |  |  |  | X |  |  |
| LHX4 |  | X |  |  |  |  |  |  |  |
| LHX5 |  | X |  |  |  |  |  |  |  |
| LIF |  |  |  |  |  | X |  |  |  |
| LIM1 |  | X |  |  |  |  |  |  |  |
| LRP8 |  | X |  |  |  |  |  |  |  |
| MANSC4 |  | X |  |  |  |  |  |  |  |
| MAP1L |  | X |  |  |  |  |  |  |  |
| MAPK14 |  | X |  |  |  |  |  |  |  |
| MASH1 |  | X |  |  |  |  |  |  |  |
| MASTL |  | X |  |  |  |  |  |  | X |
| MATH4A |  | X |  |  |  |  |  |  |  |
| MCMBP |  | X |  |  |  |  |  |  |  |
| MED21 |  | X |  |  |  |  |  |  |  |
| MEIS1 |  | X |  |  |  |  |  |  |  |
| MEOX1 |  | X |  |  |  |  |  |  |  |
| MET |  | X |  |  |  | X |  |  |  |
| METTL20 |  | X |  |  |  |  |  |  |  |
| MKKS (BBS6) |  |  |  |  |  |  |  |  |  |
| MKRN3 |  |  |  |  |  |  | X |  |  |
| MRPS35 |  | X |  |  |  |  |  | X |  |
| MSX1 |  | X |  |  |  |  |  | X |  |
| MSX2 |  | X |  |  |  |  |  |  |  |
| MTOR |  |  |  |  |  | X |  |  |  |
| MYCN |  | X |  |  |  | X |  |  |  |
| NCAM |  | X |  |  |  |  |  |  |  |
| NDN |  | X |  |  |  | X | X |  |  |
| NEUROD |  | X |  |  |  |  |  |  |  |
| NEUROG2 |  | X |  |  |  |  |  |  |  |
| NF1 |  | X |  |  |  |  |  |  |  |
| NK2 |  | X |  |  |  |  |  |  |  |
| NODAL |  | X |  |  |  |  |  |  |  |
| NOS1 |  | X |  |  |  | X |  |  | X |
| NOTCH1 |  | X |  |  |  |  |  |  | X |
| NR0B1 (DAX1) | X | X |  | X |  | X | X |  |  |
| NR5A1 |  |  |  |  |  | X |  |  |  |
| NRP1 |  | X |  |  |  | X |  |  |  |
| NRP2 |  | X |  |  |  | X |  |  | X |
| NSMF (NELF) | X | X |  | X | X | X | X | X | X |
| NSCL2 |  | X |  |  |  |  |  |  |  |
| NSMCE4A |  | X |  |  |  |  |  |  |  |
| NTN |  | X |  |  |  |  |  |  |  |
| NTN1 |  | X |  |  |  | X |  |  |  |
| OBP2B |  | X |  |  |  |  |  |  |  |
| OCT1 |  | X |  |  |  |  |  |  |  |
| OL14RD | X |  |  |  |  |  |  |  |  |
| OMP |  | X |  |  |  |  |  |  |  |
| OTUD4 | X |  |  |  |  |  | X |  |  |
| OTX2 |  | X |  |  |  | X |  | X |  |
| OVCH1 |  | X |  |  |  |  |  |  |  |
| P75NTR |  | X |  |  |  |  |  |  |  |
| PALM2 |  | X |  |  |  |  |  |  | X |
| PAX6 |  | X |  |  |  | X |  |  |  |
| PAX7 |  | X |  |  |  |  |  |  |  |
| PCDH8 |  | X |  |  |  |  |  |  |  |
| PCSK1 | X | X |  |  | X |  | X |  |  |
| PDE3A |  | X |  |  |  |  |  |  | X |
| PENK |  | X |  |  |  |  |  |  |  |
| PIT1 |  | X |  |  |  |  |  |  |  |
| PITX2 |  | X |  |  |  |  |  |  |  |
| PKNOX1 |  | X |  |  |  |  |  |  |  |
| PKNOX2 |  | X |  |  |  |  |  |  |  |
| PLEKHA5 |  | X |  |  |  |  |  |  | X |
| PLXNA1 |  | X |  |  |  | X |  |  |  |
| PLXNB1 |  | X |  |  |  | X |  |  |  |
| PNPLA6 | X |  |  |  |  |  | X | X |  |
| POLR3A |  |  |  |  |  |  | X | X |  |
| POLR3B |  |  |  |  |  |  | X | X |  |
| POMC |  | X |  |  |  |  |  |  |  |
| POU1F1 |  | X |  |  |  |  |  |  |  |
| POU5F1 |  | X |  |  |  |  |  |  |  |
| PPAPDC1A |  | X |  |  |  |  |  |  |  |
| PPFIBP1 |  | X |  |  |  |  |  |  |  |
| PREP1 |  | X |  |  |  |  |  |  |  |
| PROK2 | X | X |  | X | X | X | X | X |  |
| PROKR1 |  | X |  |  |  |  |  |  |  |
| PROKR2 | X | X |  | X | X | X | X | X | X |
| PROP1 |  | X |  |  |  |  | X |  |  |
| PTC1 |  | X |  |  |  |  |  |  |  |
| PTCH1 |  | X |  |  |  |  |  |  |  |
| PTCH2 |  | X |  |  |  |  |  |  |  |
| PTHLH |  | X |  |  |  |  |  |  |  |
| PTX1 |  | X |  |  |  |  |  |  |  |
| RAB18 |  |  |  |  |  |  | X |  |  |
| RAB3GAP1 |  |  |  |  |  |  | X |  |  |
| RAB3GAP2 |  |  |  |  |  |  | X |  |  |
| RAC |  | X |  |  |  |  |  |  |  |
| RBM28 |  |  |  |  |  |  | X |  |  |
| RD3 |  | X |  |  |  |  |  |  | X |
| RELN |  | X |  |  |  | X |  |  |  |
| REP15 |  | X |  |  |  |  |  |  |  |
| RNF216 | X |  |  |  |  |  | X | X |  |
| ROBO3 |  |  |  |  |  | X |  |  |  |
| RTP1 |  | X |  |  |  |  |  |  |  |
| RTP2 |  | X |  |  |  |  |  |  |  |
| RUNX1 |  | X |  |  |  |  |  |  |  |
| RUNX2 |  | X |  |  |  |  |  |  |  |
| RUNX3 |  | X |  |  |  |  |  |  |  |
| SCAF1 (SRA1) |  |  |  |  |  |  | X |  |  |
| SCGB1c1 |  | X |  |  |  |  |  |  |  |
| SCIP |  | X |  |  |  |  |  |  |  |
| SDR16C5 |  | X |  |  |  |  |  |  |  |
| SEC14L3 |  | X |  |  |  |  |  |  |  |
| SEC23IP |  | X |  |  |  |  |  |  |  |
| SEMA3A | X | X |  | X | X | X | X | X | X |
| SEMA3E |  |  |  |  |  |  | X |  |  |
| SEMA4D |  | X |  |  |  | X |  |  |  |
| SEMA7A | X |  |  |  |  | X | X | X |  |
| SFRP1 |  | X |  |  |  |  |  |  |  |
| SFRP5 |  | X |  |  |  |  |  |  |  |
| SH2D3C |  | X |  |  |  |  |  |  |  |
| SHH |  | X |  |  |  |  |  |  |  |
| SIX3 |  | X |  |  |  |  |  |  |  |
| SIX6 |  | X |  |  |  | X |  |  |  |
| SLC17A6 |  | X |  |  |  |  |  |  |  |
| SLCO1C1 |  | X |  |  |  |  |  |  |  |
| SLIT2 |  |  |  |  |  | X |  |  |  |
| SMAD3 |  | X |  |  |  |  |  |  |  |
| SMAD4 |  | X |  |  |  |  |  |  |  |
| SMAD5 |  | X |  |  |  |  |  |  |  |
| SMO |  | X |  |  |  |  |  |  |  |
| SOX10 | X |  |  |  | X | X | X | X |  |
| SOX2 |  | X |  |  |  | X | X |  |  |
| SOX3 |  | X |  |  |  |  | X |  |  |
| SP8 |  | X |  |  |  |  |  |  |  |
| SPRY2 |  |  |  |  |  | X |  |  |  |
| SPRY4 |  | X |  | X |  | X | X | X | X |
| ST8SIA1 |  | X |  |  |  |  |  |  |  |
| STIL |  | X |  |  |  |  |  |  |  |
| STK38L |  | X |  |  |  |  |  |  |  |
| STMN1 |  | X |  |  |  |  |  |  |  |
| STS |  |  |  |  |  | X |  |  |  |
| STUB1 |  |  |  |  |  |  | X |  |  |
| SUFU |  | X |  |  |  |  |  |  |  |
| SYCP1 |  | X |  |  |  |  |  |  |  |
| TAC3 | X | X |  | X | X | X | X | X | X |
| TACR3 | X | X |  | X | X | X | X | X | X |
| TAG-1 |  | X |  |  |  |  |  |  |  |
| TBC1D20 |  |  |  |  |  |  | X |  |  |
| TBR1 |  | X |  |  |  |  |  |  |  |
| TBX2 |  | X |  |  |  |  |  |  |  |
| TGFB |  | X |  |  |  |  |  |  |  |
| TLE4 |  |  |  |  |  | X |  |  |  |
| TM7SF3 |  | X |  |  |  |  |  |  |  |
| TMTC1 |  | X |  |  |  |  |  |  |  |
| TRAPPC9 |  | X |  |  |  |  |  |  | X |
| TRIM25 |  | X |  |  |  |  |  |  |  |
| TRIM32 (BBS11) |  |  |  |  |  |  | X |  |  |
| TSPAN11 |  | X |  |  |  |  |  |  | X |
| TTC8 (BBS8) |  |  |  |  |  |  | X |  |  |
| TUBB2A |  | X |  |  |  |  |  |  |  |
| TUBB2B |  | X |  |  |  |  |  |  |  |
| TUBB3 |  | X |  |  |  |  | X |  |  |
| TYRO3 |  |  |  |  |  | X |  |  |  |
| UMODL1 |  | X |  |  |  |  |  |  |  |
| WDR11 | X | X |  | X | X | X | X | X |  |
| WNT11 |  | X |  |  |  |  |  |  |  |
| WNT8b |  | X |  |  |  |  |  |  |  |
| YME1L1 |  | X |  |  |  |  |  |  |  |
| ZFP423 |  | X |  |  |  |  |  |  |  |
